# Latin American Pain Federation position paper on appropriate opioid use in pain management

**DOI:** 10.1097/PR9.0000000000000730

**Published:** 2019-05-21

**Authors:** João Batista Santos Garcia, Maria Patricia Gomez Lopez, Guilherme Antonio Moreira Barros, Héctor G Molina Muñiz, Marisol Ahumada Olea Ahumada Olea, Patricia Bonilla, Elizabeth Díaz Pérez de Valtolina, Daniel Neves Forte, María del Rocío Guillén Nuñez, Bethania Martinez del Villar, Nicolas Sarria, José Manuel Barrientos Peñaloza, Durval Campos Kraychete, Eduardo Grossmann, André Filipe Junqueira dos Santos, Debora Brigitte Martineau Arteaga, Manoel Jacobsen Teixeira

**Affiliations:** a Federal University of Maranhao, São Luis, Brazil; b Universidad Nacional, Bogota, Colombia; c Botucatu Medical School, Sao Paulo, Brazil; d Centro de Intervencionismo en Dolor, Guatemala; e Hospital Clínico La Florida, Santiago, Chile; f Universidad Técnica Particular de Loja, Ecuador; g Instituto Nacional de Enfermedades Neoplásicas, Lima, Peru; h Hospital Sirio-Libanes, Sao Paulo, Brasil; i Instituto Nacional de Cancerología, Mexico City, Mexico; j Santo Domingo, Dominican Republic; k Universidad Catolica de Cordoba, Argentina; l Hospital Materno Infantil, Caja Nacional de Salud, La Paz, Bolivia; m Federal University of Bahia, Salvador, Brazil; n Federal University of Rio Grande do Sul, Porto Alegre, Brazil; o Instituto Oncológico de Ribeirão Preto/Grupo Oncoclinicas, Sao Paulo, Brazil; p Universidad Central del Ecuador, Quito, Ecuador; q Universidade de Sao Paulo(USP), Sao Paulo, Brazil

## 1. Introduction

Improper pain treatment is a worldwide public health issue.^5,8^ Although scientific evidence supports the safe use of opioids,^2^ there is still significant reluctance around the use of these analgesics. The most significant problem for appropriate pain management has been the negative perception of these analgesics, known as opiophobia.^1,7^

The “opioid crisis,” characterised by the prolonged and indiscriminate use of prescription opioids seen in some countries, has led to high addiction and mortality rates. This has had a profoundly negative impact on pain management.^3,7,8^ Furthermore, in Latin America and for most of the world's population, access to and availability of these medicinal products are still inadequate and lead to unnecessary suffering as a result.^6,7^

Inadequate pain management in Latin American countries may be worsened by the “opioid crisis” that other regions of the world are undergoing,^6,7^ which is just one of the many reasons that warrants the drafting of this document. Latin American Federation of IASP Chapters (FEDELAT) convened a group of experts from the region (from Mexico to Chile, including the Caribbean) in São Paulo city (Brazil) to prepare a position paper on appropriate opioid use in chronic pain. All recommendations are based on the group's analysis of the needs and particularities of the region. It is hoped that this regional position paper will improve regulations and pain management, as well as prevent abuse and misuse of these medicinal products.

Most people dying of terminal chronic diseases in developing countries do not have access to controlled medicinal products for pain management. Worldwide, around 25 million people died in 2015 of terminal chronic diseases. Approximately 80% of these were in developing countries, and many of them died suffering from pain.^7^

The situation in Latin America regarding access to and availability of opioids is still limited and is below 100 statistically defined daily doses. Countries with the lowest recorded consumption include Guatemala, Ecuador, and Bolivia. In Chile, Argentina, Colombia, Brazil, and Uruguay, opioid consumption has been successfully raised to 200 statistically defined daily doses. However, this figure is still not sufficient for adequate pain management.^6,7^

There is a lack of publications which have looked at opioid abuse by pain patients in Latin America. There are some data about the prevalence of prescription opioid exposure at least once in a person's life, with an incidence of around 1%. Problems with abuse occur mainly with alcohol, marijuana, and cocaine.^4,7^ Thus, the perceived abuse risk of opioids in Latin America is very low.

## 2. Suggestions for Latin America regarding opioid use

### 2.1. Education

Promote training in the safe use of opioid analgesics based on protocols and on scientific evidence.

The creation of specific platforms on the website of the FEDELAT and of the Latin American Association for Palliative Care (ALCP) has been suggested, which would contain virtual courses with updated information on indications, management, and precautions for the use of opioids.

Promote local training for health care professionals and patients in every country led by scientific and academic organisations in cooperation with FEDELAT and ALCP. Develop educational materials for patients and the general community on appropriate opioid use and the risk of abuse.

### 2.2. Advocacy and public policy

Raise awareness among decision-makers about the need to create pain treatment programmes and promote a balance between sufficient access to controlled substances for medical and scientific purposes and avoiding opioid misuse.

Create national guidelines based on international recommendations on appropriate use, such as medicinal product selection, dose calculation, opioid rotation, management of high-risk patients, and treatment monitoring.

### 2.3. Digital prescriptions

Promote the creation of a digital registration system which can be used to prescribe opioids, monitor the risk of improper use, and facilitate patient access, disease diagnosis, daily doses, and monitoring of medicinal product stock, and time and duration of prescriptions (ideally for 1 month).

This system would also contribute to reliable opioid medicinal product planning and acquisition required by every country.

It is recommended that weaker opioids should be available by prescription with a copy kept by the pharmacist, to have more control over those medicinal products that may be potentially abused. Strong opioids must follow international regulations governing narcotic drugs.

### 2.4. Statistics

Promote national systems that register opioid statistics taken from population data, hospital data, and private consultations that will provide official data on the importation, consumption, and distribution of opioids for medical use.

### 2.5. Multidisciplinary monitoring

Training is recommended for pain and palliative care units in every country, consisting of a multidisciplinary and interdisciplinary team that ensures appropriate assessment, diagnosis, multimodal therapy, and patient follow-up to minimise the dose and duration of use of prescribed opioids, particularly in patients with nononcological chronic pain.

### 2.6. Interorganisational cooperation

Scientific organisations such as FEDELAT, ALCP, and the Latin American national divisions of the International Association for the Study of Pain (IASP) should develop common plans to improve opioid availability and accessibility, endeavouring to minimise their abuse and misuse.

### 2.7. Conflicts of interest

Support governmental bodies in the implementation of conflicts of interest policies concerning undue influence from all for-profit bodies in tendering, procurement, and marketing of opioid medicinal products.

### 2.8. Conclusions

Public health organisations in Latin America should improve their ability to produce statistics to facilitate greater understanding of the actual situation in the region. It is important that organisations involved in the education, regulation, and marketing of opioid analgesics work together to encourage proper use and monitoring of these medicinal products. These measures could lead to a decrease in opiophobia, which has risen in Latin America.

The opioid crisis in Latin America is different to the one currently unfolding in the United States, Canada, and other developed countries. Our current crisis is one of undertreatment and suffering.

## Disclosures

The authors have no conflict of interest to declare.
